# Timing and efficacy of doxycycline in macrolide-resistant *Mycoplasma pneumoniae* pneumonia in children: a single-center retrospective study

**DOI:** 10.3389/fpubh.2026.1727627

**Published:** 2026-02-04

**Authors:** Shaoying Liu, Lijun Zhang, Lei Dai, Deyuan Li

**Affiliations:** 1 Department of Pediatrics, West China Second University Hospital, Sichuan University, Chengdu, Sichuan, China; 2 Key Laboratory of Birth Defects and Related Disease of Women and Children (Sichuan University), Ministry of Education, Chengdu, Sichuan, China; 3 Department of Biotherapy, Cancer Center and State Key Laboratory of Biotherapy, West China Hospital, Sichuan University, Chengdu, Sichuan, China

**Keywords:** antibiotics, children, electronic bronchoscopy, hormones, oxygen therapy, severe *Mycoplasma pneumoniae* pneumonia, treatment

## Abstract

**Background:**

Outbreaks of macrolide-resistant *Mycoplasma pneumoniae* (MRMP) in children have posed ongoing treatment challenges. This study aimed to assess the efficacy of doxycycline in treating MRMPP at different time points, offering insights for public health strategies.

**Methods:**

We retrospectively analyzed children with MRMPP hospitalized between September 2022 and February 2024. They were divided into three main groups based on antibiotic use: (1) those who received azithromycin only (AZI group); (2) those who received doxycycline only (DOX group); (3) those who received azithromycin followed by doxycycline (ATD group), divided into two subgroups according to the duration of azithromycin use: azithromycin use < = 3-day subgroup (ATD1) and azithromycin use > 3-day subgroup (ATD2). Oxygen therapy, electronic bronchoscopy, hormones and gamma globulin were also recorded. Length of hospital stay and duration of fever were used as outcome measures for comparative analyses. Propensity score matching (PSM) analysis was used to adjust the score.

**Results:**

312 eligible children were identified. The DOX group had the highest 48-h fever reduction rate (80%). The AZI group had a higher proportion of oxygen (77%) and e-bronchoscopy (75%) use, and the ADT group had a higher proportion of hormone use (39%). After adjustment for PSM, oxygen use remained higher in the ATD2 group than in the DOX+ATD1 group (*p* = 0.026).

**Conclusions:**

Early use of doxycycline in treating MRMPP reduces reliance on adjuvant therapies, thus easing the burden on healthcare resources.

## Graphical Abstract

**Figure:**
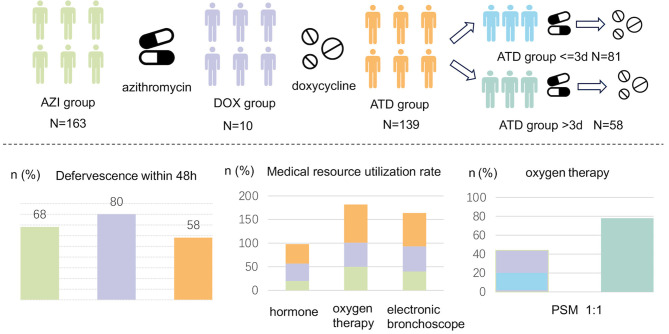
Study groups and key outcomes.

## 1 Introduction

In the past 2 years, outbreaks of MRMP infections have occurred in mainland China and even worldwide. Among the variants associated with MRMP infection, A2063G is the most common variant (96.8%) (1). Two main types of clonal strains, EC1 and EC2, exist in China (2), with the EC1 type predominating in the past, but since the outbreak of MRMP, there has been a trend toward the EC2 type (3). The mechanism by which this phenomenon occurs is unclear. Nevertheless, Oishi et al. (4) concluded that the misuse of macrolide antibiotics may be one of the reasons for an azithromycin exposure test.

It has been shown (5) that mutant strains induced by 14- and 15-membered macrolides are generally resistant to all macrolide antibiotics, whereas mutant strains induced by 16-membered macrolides partially maintain sensitivity to 14- and 15-membered macrolides antibiotics. This may indicate that drug-resistant gene-positive *Mycoplasma pneumoniae* may be partially susceptible to macrolides. The treatment of MRMPP remains controversial, and delay in effective treatment is one of the significant risk factors for extrapulmonary complications in MRMPP (6). Nowadays, a comprehensive management approach is commonly adopted for MRMPP, with antibiotic therapy as the mainstay in combination with other symptomatic supportive treatments. This study aims to analyse the efficacy of doxycycline in the treatment of MRMPP in children at different times.

## 2 Methods

### 2.1 Study population and groups

This was a retrospective cohort study conducted at West China Second Hospital of Sichuan University, a tertiary pediatric referral center that admits patients from multiple provinces in Southwest China. All hospitalized children diagnosed with MRMP infection from September 2022 to February 2023 were included in this study. Eligible patients were required to have clinical manifestations consistent with *M. pneumoniae* pneumonia, including fever, cough, and radiographic evidence of pneumonia. MRMP infection was confirmed by a positive nucleic acid test for *M. pneumoniae* combined with identification of 23S rRNA point mutations (A2063G or A2064G) indicating macrolide resistance.

Exclusion criteria were (1) automatic discharge; (2) presence of immunodeficiency; (3) severe organ dysfunction (cardiac: LVEF < 35% or NYHA Class III-IV; hepatic: Child-Pugh B/C or ALT/AST >3 × ULN with bilirubin >2 × ULN; renal: eGFR < 30 ml/min/1.73 m^2^ or dialysis); and (4) mixed other viral or bacterial infections.

The antibiotics used for treatment and the adjuvant treatment modalities were decided by the child's attending physician, and according to the instructions for the use of the medicines, we followed azithromycin 10 mg/kg once daily and doxycycline 2 mg/kg every 12 h. All children younger than 8 years of age were given consent by a pharmacist in consultation before doxycycline was administered. We divided the children into three groups according to the use of azithromycin and doxycycline: (1) those who received azithromycin only (AZI group); (2) those who received doxycycline only (DOX group); and (3) those who received azithromycin followed by doxycycline (ATD group). Among them, the ATD group was further divided into two subgroups based on azithromycin treatment duration: ≤ 3 days (ATD1) and >3 days (ATD2), according to the 2025 Chinese pediatric MPP guideline (7), which defines macrolide-unresponsive cases as those showing no improvement after 72 h of macrolide therapy.

### 2.2 Data collection

This was a retrospective study in which we collected information from the cases of children. Basic information included age, sex, presence of fever, duration of fever before treatment, duration of fever after starting treatment, wheezing and extrapulmonary complications. The laboratory evaluation consisted of complete blood count with measurements of white blood cell count, hemoglobin concentration, and platelet count, along with high-sensitivity C-reactive protein (hs-CRP) levels. Liver function assessment included aspartate aminotransferase (AST), alanine aminotransferase (ALT), and lactate dehydrogenase (LDH) activities. Microbiological analysis comprised sputum culture and molecular detection of *Mycoplasma pneumoniae* macrolide resistance genes from pharyngeal swab specimens, specifically nucleic acid amplification testing for *Mycoplasma pneumoniae* identification and characterization of A2063G/A2064G resistance mutations in the 23S rRNA gene. There were also imaging performances of the child. The child's adjunctive therapies other than antibiotics, including oxygen therapy, electronic bronchoscopy, intravenous methylprednisolone (0.5–1 mg/kg/day) and intravenous immunoglobulin (IVIG), were also collected.

### 2.3 Endpoints

The primary endpoints of this study were length of hospital stay and time to defervescence after initiation of antibiotic therapy. The secondary endpoints included the use of supportive and adjunctive therapies, specifically oxygen supplementation, intravenous methylprednisolone, electronic bronchoscopy, and IVIG during hospitalization.

Length of hospital stay was defined as the number of days from hospital admission to discharge.

Time to defervescence was assessed using three predefined measures: overall time to defervescence, defervescence within 24 h, and defervescence within 48 h after antibiotic initiation. Defervescence was defined as an axillary body temperature < 37.5 °C maintained for at least 24 consecutive hours without the use of antipyretic agents. Overall time to defervescence was calculated as the total number of days from the initiation of antibiotic treatment to the first day meeting the defervescence criteria. Defervescence within 24 and 48 h were recorded as binary outcomes based on whether the temperature normalization occurred within the respective time windows.

Oxygen therapy was defined as the administration of supplemental oxygen via nasal cannula, mask, or other oxygen delivery devices due to hypoxemia or respiratory distress.

All endpoints were extracted from electronic medical records and were determined prior to statistical analysis.

### 2.4 Statistical analysis

We used the R language (version 4.4.1) to perform all statistical analyses. *p* < 0.05 was considered statistically significant. Because we collected data with missing values in AST and ALT, which were all non-normally distributed, the median-filling method was used to supplement the missing values. The CBCgrps2.8-package was used to compare the baseline characteristics of the two groups, where categorical variables were analyzed using the chi-square test. Measurements that were not normally distributed were analyzed using the Mann-Whitney *U* test. Given the observational nature of the study and the potential confounding by indication in antibiotic selection, propensity score matching (PSM) was performed to evaluate the effect of early vs. delayed use of doxycycline. For this analysis, patients in the DOX group and ATD1 group were combined as the early doxycycline group, while patients in the ATD2 group constituted the delayed doxycycline group. The propensity score was estimated using a multivariable logistic regression model. Covariates included age, sex, wheezing, presence of extrapulmonary complications, baseline laboratory parameters (white blood cell count, hemoglobin, platelet count, high-sensitivity C-reactive protein, alanine aminotransferase, aspartate aminotransferase, and lactate dehydrogenase), and radiological findings (lung consolidation, lung exudation, pleural thickening, pleural effusion, lung patchy shadows, and mosaic signs). Patients were matched in a 1:1 ratio using nearest-neighbor matching without replacement, with a caliper width of 0.02.

## 3 Result

### 3.1 Basic characteristics

According to our inclusion and exclusion criteria, there were 312 final eligible children. Table 1 compares the baseline characteristics among the AZI, DOX, ATD1, and ATD2 groups. Overall, there were no significant differences in most clinical features, laboratory parameters, or imaging findings. The mean age of the children was approximately 6–7 years, with similar gender distribution across groups. Clinical manifestations such as fever, cough, and wheezing were comparable, with *p*-values well above 0.05. Laboratory results, including blood routine indices (WBC, HGB, PLT), inflammatory markers (hs-CRP), and liver function tests (ALT, AST), showed no significant differences among groups. Lung imaging findings were largely similar, with the proportion of lung consolidation ranging from 67 to 80% across groups. The only exception was pleural effusion, which showed a statistically significant difference (*p* = 0.048), while other imaging features such as pleural thickening, lung patch, mosaic signs, and bronchial occlusion did not differ significantly.

**Table 1:** Comparison of characteristics between AZI, DOX, ATD2 and ATD2 groups.

**Factors**	**AZI (*n* = 163)**	**DOX (*n* = 10)**	**ATD1 (*n* = 81)**	**ATD2 (*n* = 58)**	***p*-Value**
Age, mean ± SD	6.39 ± 2.6	6.39 ± 2.72	7.2 ± 2.45	6.47 ± 2.88	0.136
Male, *n* (%)	82 (50)	3 (30)	34 (42)	32 (55)	0.273
Fever, *n* (%)	160 (98)	10 (100)	80 (99)	55 (95)	0.442
Duration of fever before treatment, median (Q1, Q3)	6 (4, 8)	6.5 (5, 8)	6 (4, 7)	5 (4, 7)	0.057
Cough, *n* (%)	163 (100)	10 (100)	81 (100)	58 (100)	1
Wheezing, *n* (%)	15 (9)	0 (0)	8 (10)	7 (12)	0.805
Extrapulmonary complications, *n* (%)	11 (7)	1 (10)	3 (4)	5 (9)	0.451
WBC, median (Q1, Q3)	7.2 (5.8, 9.5)	7.2 (6.35, 8.05)	7.3 (5.9, 9.1)	7.16 (5.93, 8.78)	0.982
HGB, median (Q1, Q3)	121 (114, 129.5)	132 (124.25, 134.25)	123 (116, 129)	121 (114.25, 129)	0.082
PLT, median (Q1, Q3)	266 (219, 376)	243 (207.25, 262)	275 (217, 336)	306.5 (228, 381.25)	0.555
hs-CRP, median (Q1, Q3)	13.1 (3.85, 26.2)	10.35 (5.23, 28.98)	14.7 (5.4, 41.2)	11.1 (2.85, 20.95)	0.177
ALT, median (Q1, Q3)	17 (13, 23)	18.5 (14.25, 21.25)	18 (13, 24)	16 (13, 25)	0.927
AST, median (Q1, Q3)	32 (28, 38)	35 (31.25, 40.75)	32 (26, 38)	32 (27, 42)	0.539
LDH, median (Q1, Q3)	318 (275, 384)	344 (284.5, 406)	305 (255, 344)	313 (264, 378)	0.318
Consolidation of the lung, *n* (%)	112 (69)	8 (80)	62 (77)	39 (67)	0.52
Lung exudation, *n* (%)	2 (1)	0 (0)	1 (1)	1 (2)	1
Pleural thickening, *n* (%)	34 (21)	5 (50)	18 (22)	16 (28)	0.166
Pleural effusion, *n* (%)	33 (20)	1 (10)	26 (32)	8 (14)	0.048
Lung patch, *n* (%)	119 (73)	5 (50)	49 (60)	42 (72)	0.112
Mosaic signs, *n* (%)	4 (2)	0 (0)	3 (4)	3 (5)	0.651
Bronchial occlusion, *n* (%)	6 (4)	0 (0)	1 (1)	2 (3)	0.742

AZI, azithromycin only group; DOX, doxycycline only group; ATD1, azithromycin followed by doxycycline group and azithromycin use < = 3-day; ATD2, azithromycin followed by doxycycline group and azithromycin use > 3-day; WBC, white blood cell; HGB, hemoglobin level; PLT, platelet count; hs-CRP, C-reactive protein; ALT, alanine aminotransferase; AST, aspartate aminotransferase; LDH, lactate dehydrogenase.

### 3.2 Comparison of treatments and efficacy

This study compared the three groups for important adjunctive therapies (oxygen therapy, electronic bronchoscopy, intravenous methylprednisolone and intravenous IVIG) other than antibiotics. We assessed the efficacy of the three groups by using the length of hospitalization and the duration of fever after the start of treatment as the outcome indicators, as shown in Table 2. There was no significant difference in the length of hospitalization (*p* = 0.282). However, there was a significant difference in the duration of fever after drug treatment (*p* < 0.001), and we can get that the ATD group had the slowest return of temperature to normal after antibiotic use, and the median duration of fever was the same after doxycycline alone or azithromycin alone. Although there was no significant difference in the percentage of temperature reduction to normal within 48 h, the percentage of temperature reduction to normal within 48 h was 80% in the DOX group and 68% in the AZI group, which was slightly lower than the DOX group, while the ATD group remained the lowest at 58%. As can be seen in Table 2, there was a significant difference in other treatments among the three groups, except for the use of gammaglobulin. In the use of hormones, the ATD group was significantly higher than the AZI and DOX groups (*p* < 0.001), with 39% of the children having used hormones. The use of oxygen therapy and bronchoscopy was higher in the AZI group (*p* = 0.012 and 0.004, respectively), both being more than 70%, whereas the DOX group continued to have the lowest percentage of oxygen therapy and bronchoscopy.

**Table 2:** Comparison of adjuvant treatment methods of AZI, DOX and ATD groups.

**Factors**	**AZI (*n* = 163)**	**DOX (*n* = 10)**	**ATD (*n* = 139)**	***p*-Value**
Total hospital days, median (Q1, Q3)	9 (7, 10)	10.5 (8.25, 11)	8 (7, 11)	0.282
Duration of fever after treatment, median (Q1, Q3)	1 (0, 3)	1 (0, 1.75)	2 (0, 5)	0.035
Defervescence within 48 h, *n* (%)	111 (68)	8 (80)	80 (58)	0.104
Hormone, *n* (%)	29 (18)	2 (20)	54 (39)	< 0.001
Oxygen therapy, *n* (%)	125 (77)	5 (50)	88 (63)	0.012
Electronic bronchoscope, *n* (%)	122 (75)	4 (40)	84 (60)	0.004
Immunoglobulin, *n* (%)	9 (6)	0 (0)	7 (5)	1

AZI, azithromycin only group; DOX, doxycycline only group; ATD, azithromycin followed by doxycycline group.

### 3.3 Evaluation of the efficacy of doxycycline at different stages

We divided ATD into the ATD1 group and the ATD2 group. As can be seen in Table 3, there was no significant difference in the outcome indicators, i.e., hospitalization time, 24- and 48-h defervescence rates and duration of fever in the three groups. However, in the DOX group, the defervescence rates at 24- and 48- h were 70 and 80%, respectively. There was a significant difference in oxygen therapy, which was significantly higher in the ATD2 group than in the DOX and ATD1 groups (*p* < 0.001), accounting for 81% of the number of people in the ATD2 group, compared to 50 and 51% in the other two groups, respectively. The rate of bronchoscopic was 71% in the ATD2 group and 40 and 53% in the DOX and ATD1 groups, and although this was not a significant difference (*p* = 0.052), it was still evident that a higher proportion of ATD2 used electronic bronchoscopic lavage.

**Table 3:** Assessment of the efficacy of doxycycline at different periods.

**Factors**	**DOX (*n* = 10)**	**ATD1 (*n* = 81)**	**ATD2 (*n* = 58)**	***p*-Value**
Total hospital days, median (Q1, Q3)	10.5 (8.25, 11)	9 (7, 12)	8 (7, 10)	0.241
Fever, *n* (%)	10 (100)	80 (99)	55 (95)	0.477
Defervescence within 24 h, *n* (%)	7 (70)	39 (48)	25 (43)	0.301
Defervescence within 48 h, *n* (%)	8 (80)	48 (59)	32 (55)	0.365
Duration of fever, median (Q1, Q3)	8 (5.25, 10)	8 (6, 10)	8 (6, 9.75)	0.674
Hormone, *n* (%)	2 (20)	30 (37)	24 (41)	0.457
Oxygen therapy, *n* (%)	5 (50)	41 (51)	47 (81)	< 0.001
Electronic bronchoscope, *n* (%)	4 (40)	43 (53)	41 (71)	0.052
Immunoglobulin, *n* (%)	0 (0)	5 (6)	2 (3)	0.817

DOX, doxycycline only group; ATD1, azithromycin followed by doxycycline group and azithromycin use < = 3-day; ATD2, azithromycin followed by doxycycline group and azithromycin use > 3-day.

### 3.4 Evaluation of the efficacy of doxycycline use at different stages after matching analyses using the PSM

We divided the DOX+ATD1 group into the early doxycycline use group. The ATD2 group is the late doxycycline use group. These two groups were compared to assess the efficacy of doxycycline use at different stages. We used the PSM propensity matching score for 1:1 matching and set the caliper value to 0.02 (Table 4).

**Table 4:** Assessment of the efficacy of doxycycline use at different periods after PSM matched analysis.

**Factors**	**DOX+ATD1 (*n* = 27)**	**ATD2 (*n* = 27)**	***p*-Value**
Age, mean ± SD	7.3 ± 2.3	7.07 ± 2.57	0.735
Total hospital days, median (Q1, Q3)	8 (6, 10.5)	9 (7, 10.5)	0.438
Lung patch, *n* (%)	19 (70)	16 (59)	0.569
Mosaic signs, *n* (%)	1 (4)	0 (0)	1
Bronchial occlusion, *n* (%)	1 (4)	1 (4)	1
Fever, *n* (%)	27 (100)	26 (96)	1
Duration of fever before treatment, Median ± SD	5.63 ± 2.06	4.89 ± 2.99	0.295
Duration of fever after treatment, Median (Q1, Q3)	3 (0, 5.5)	3 (1, 5)	1
Azithromycin usage time, median (Q1, Q3)	3 (2, 3)	5 (5, 6)	< 0.001
Hormone, *n* (%)	9 (33)	13 (48)	0.406
Oxygen therapy, *n* (%)	12 (44)	21 (78)	0.026
Electronic bronchoscope, *n* (%)	11 (41)	17 (63)	0.173
Immunoglobulin, *n* (%)	1 (4)	1 (4)	1

DOX, doxycycline only group; ATD1, azithromycin followed by doxycycline group and azithromycin use < = 3-day; ATD2, azithromycin followed by doxycycline group and azithromycin use > 3-day.

Baseline characteristics, including age, radiological findings, presence of fever, and duration of fever before treatment, were well-balanced between the early doxycycline group (DOX+ATD1) and the late doxycycline group (ATD2). No significant differences were observed between the two groups in total length of hospitalization or duration of fever after treatment initiation (all *p* > 0.05). The distribution of imaging features, including lung consolidation, mosaic attenuation, and bronchial occlusion, was also comparable between groups. However, patients in the late doxycycline group required oxygen therapy more frequently than those in the early doxycycline group (78 vs. 44%, *p* = 0.026). In addition, the proportion of patients receiving systemic corticosteroids was higher in the late doxycycline group (48 vs. 33%), although this difference did not reach statistical significance. Other supportive treatments, including bronchoscopy and immunoglobulin administration, showed no significant differences between the two groups.

## 4 Discussion

Our study found few significant differences in the epidemiological characteristics of the different groups, which may suggest that the severity of the disease was similar among the children in the different groups. This is understandable, as an epidemiological survey in Japan (8) showed that infections caused by MRMPP also did not show significant differences in clinical manifestation and incidence among patients of different age groups. Although pleural effusion showed a statistically significant difference (*p* = 0.048), other clinical and laboratory indicators were comparable, indicating that this isolated finding may have limited clinical significance. Notably, while the ATD1 subgroup had a higher proportion of pleural effusion, children in the ATD2 subgroup required more supportive therapies such as oxygen supplementation, corticosteroids, and IVIG. This discrepancy suggests that pleural effusion alone may not adequately reflect subsequent disease progression or treatment response. Taken together, the overall comparability of baseline disease severity across groups supports the validity of subsequent between-group comparisons. In this context, the observed differences in supportive treatment requirements are unlikely to be fully explained by baseline disease severity alone and may be associated with differences in treatment timing. Notably, children who received earlier doxycycline intervention tended to require fewer additional therapies, suggesting a potential clinical benefit of timely administration of effective non-macrolide antibiotics in attenuating disease progression and inflammatory burden. This interpretation is further supported by the propensity score–matched analysis, which was designed to balance measured baseline confounders and to explore the association between doxycycline initiation timing and clinical outcomes in the DOX+ATD1 and ATD2 groups.

In this study, the duration of fever after treatment was significantly shorter in pediatric patients treated directly with doxycycline. This is similar to another study in which patients with MRMPP showed significantly shorter fever and hospitalization time with oral doxycycline (9). Fever duration, hospitalization days, and chest radiograph improvement are commonly used outcomes in studies of pediatric MRMP pneumonia and are clinically meaningful, as they reflect the speed of symptom resolution, disease severity, and short-term recovery, all of which are highly relevant for guiding treatment decisions in real-world clinical practice. It is noteworthy that, despite comparable durations of fever between the AZI group and the DOX group in this high MRMP prevalence setting, this observation may be attributed to the natural progression of the disease and the therapeutic characteristics. At enrollment, patients had experienced fever for a median duration of 6 days, indicating most cases had progressed to mid-late disease stages, during which both antimicrobial agents demonstrated comparable antipyretic effects through modulation of inflammatory responses. However, improvement in respiratory symptoms exhibited relative delay, as evidenced by significantly higher rates of oxygen therapy (77 vs. 50%) and bronchoscopic intervention (75 vs. 40%) in the azithromycin group. Furthermore, the limited sample size of the primary doxycycline treatment cohort (*n* = 10) may have constrained the statistical power for between-group comparisons. These findings collectively suggest that while doxycycline demonstrates clinically meaningful antipyretic efficacy in MRMPP management, comprehensive therapeutic evaluation should incorporate multiple clinical parameters, particularly for patients with prolonged disease courses where dissociation may occur between fever resolution and pulmonary recovery.

Glucocorticoids inhibit the activation and proliferation of inflammatory cells, reduce inflammatory mediators such as ESR, CRP, and IL-6, and modulate CD4+ and CD4+/CD8+ levels (10, 11). For cytokine storms caused by severe infections, glucocorticoid shock therapy is effective in reducing lung injury and decreasing the incidence of severe *Mycoplasma pneumoniae* pneumonia by suppressing the overactive non-specific adaptive immune response (12). In the ATD group, it was observed that a higher proportion of patients (39%) received hormone therapy, and the combination of hormones and antibiotics improves therapeutic efficacy when antibiotic therapy fails to effectively control symptoms (13). Electronic bronchoscopy has also developed rapidly in recent years, and bronchoscopy facilitates direct observation, and accurate diagnosis, and is important for plastic bronchitis (14). Under electronic bronchoscopy, targeted treatment is carried out by delivering drugs directly to the lesion site, while reducing the side effects of systemic hormones and facilitating the expulsion of viscous sputum, thus improving symptoms and shortening hospitalization time (15, 16). In our study, for patients in the AZI group, the use of bronchoscopy was higher (75%). In addition, compared to the AZI1 group, the AZI2 group showed a further increase in the proportion of the application of bronchoscopic, which also suggests that the early use of doxycycline has a better therapeutic effect. Patients with MRMPP with hypoxia usually present with a longer duration of illness, a higher incidence of extrapulmonary complications, and more severe radiological abnormalities, which would further promote the use of glucocorticoids and bronchoscopy.

Oxygen therapy plays an important role in the treatment of MRMPP. *Mycoplasma pneumoniae* infections often cause lesions in small airways, and these lesions usually affect airways less than 2 to 4 mm in diameter. In these small airways, inflammation, oedema, secretion accumulation or structural changes may occur, leading to airflow limitation, which further leads to hypoxaemia and lung tissue damage in children (17). Therefore, the requirement for oxygen therapy can reflect the severity of airway involvement and respiratory compromise in MRMPP. In our study, the proportion of patients requiring oxygen therapy exceeded 50% across all treatment groups and was highest in the AZI group (77%). Importantly, after propensity score matching, the rate of oxygen therapy remained significantly higher in the late doxycycline group (ATD2) compared with the early doxycycline group (ATD1), despite comparable baseline characteristics and similar lengths of hospitalization and fever duration after treatment. This finding suggests that delayed initiation of effective antimicrobial therapy may be associated with a greater need for respiratory support, even when short-term clinical recovery indicators appear similar. IVIG can modulate the immune response by inhibiting cytokine production and neutralizing inflammatory factors, antigens and toxins. Although the efficacy of treatment with IVIG did not show a significant difference in our study, a study by Wei et al. (18) noted that IVIG may play an important role in the treatment of *Mycoplasma pneumoniae* pneumoniae in combination with viral infections, which suggests that, in specific cases, IVIG may help improve the clinical symptoms and outcome of patients.

Resistance of *Mycoplasma pneumoniae* to macrolide antibiotics mainly stems from specific point mutations on the 23S rRNA gene. These mutations alter the site on the 50S subunit of the ribosome that binds macrolides, resulting in the inability of the drug to bind effectively and inhibit bacterial protein synthesis, which in turn causes the phenomenon of drug resistance (19). Our study found that early administration of doxycycline resulted in reduced use of oxygen, hormones, bronchoscopy, and other adjunctive therapies without significant difference in hospitalization duration. No doxycycline-related adverse reactions were observed in our cohort. While traditional concerns regarding tetracyclines in young children remain, doxycycline is generally considered safer. Previous studies have shown that doxycycline is highly effective in treating azithromycin-resistant pediatric *Mycoplasma pneumoniae* pneumonia, alleviating inflammatory responses and shortening symptom duration, with a low incidence of short-term adverse effects (9). For children aged 8 years and older, tetracyclines may be considered as a first-line treatment option (20). Although azithromycin demonstrated some efficacy in treatment, it increased the patient's dependence on oxygen therapy. In addition, the frequency of hormone use and electronic bronchoscopy increased. However, there are some limitations of this study that deserve to be considered and addressed in future studies. First, as a retrospective study, although we compared baseline characteristics across groups to minimize confounding, residual confounding by unmeasured factors cannot be fully excluded. Second, the sample size of the DOX group was small, which may have limited the generalisability and statistical significance of our results. I addition, the limited duration of follow-up prevented a comprehensive assessment of the long-term safety and potential adverse effects of doxycycline treatment, particularly regarding outcomes such as dental or enamel changes in young children. Some multicentre prospective designs are needed in the future to increase the sample size. Meanwhile, conducting further long-term follow-ups will help us to understand the long-term effects and safety of doxycycline treatment more comprehensively.

## 5 Conclusion

Our study highlights the significant potential of early doxycycline treatment in managing MRMPP. The results demonstrate that early administration effectively alleviates key clinical symptoms, such as fever and hypoxemia, and reduces patients' reliance on adjuvant treatments like oxygen therapy, corticosteroids, and bronchoscopy. Furthermore, doxycycline shows promise in controlling lung lesion progression, providing a more effective treatment option. This approach not only benefits individual patient outcomes but also has the potential to ease the burden on healthcare systems and improve public health management by reducing the need for intensive interventions. Given the high MRMP prevalence in many Asian regions and the current lack of evidence in low-prevalence countries, our findings may offer valuable reference data for future clinical applications across diverse settings.

## Data Availability

The original contributions presented in the study are included in the article/Supplementary material, further inquiries can be directed to the corresponding author.
