# Supplementary material for: Timing and efficacy of doxycycline in macrolide-resistant Mycoplasma pneumoniae pneumonia in children: a single-center retrospective study
Source: Front Public Health. 2026 Feb 4;14:1727627. doi: 10.3389/fpubh.2026.1727627 (PMC12913422; doi:10.3389/fpubh.2026.1727627)
Supplement: Supplementary file 1 [file Data_Sheet_1.pdf]

diagnose	age	sex	Total_hospita	cough	Wheezing	Extrapulmona
0	850	1	10	1	0	1
0	525	1	6	1	0	0
0	425	0	6	1	0	1
0	475	1	7	1	0	0
0	642	0	4	1	0	0
0	558	1	11	1	0	0
0	667	1	7	1	0	0
0	292	1	12	1	0	0
0	575	1	17	1	0	0
0	442	1	6	1	0	0
0	817	1	5	1	0	0
0	400	1	8	1	0	0
0	016	0	12	1	1	0
0	283	1	9	1	0	0
0	333	1	8	1	0	0
0	917	0	7	1	0	0
0	375	0	7	1	0	0
0	408	1	9	1	0	0
0	558	1	7	1	0	0
0	617	1	8	1	0	0
0	442	0	7	1	0	0
0	650	0	5	1	0	0
0	975	0	15	1	0	1
0	592	1	8	1	0	0
0	267	0	7	1	0	0
0	475	0	23	1	1	0
0	550	1	10	1	0	0
0	842	1	11	1	0	0
0	750	0	11	1	0	0
0	683	0	5	1	0	0
0	125	0	11	1	0	1
0	908	0	6	1	0	1
0	317	1	7	1	0	0
0	742	0	9	1	0	0
0	925	0	9	1	0	1
0	825	0	5	1	0	0
0	950	1	9	1	0	0
0	475	0	13	1	1	0
0	825	0	13	1	0	0
0	975	0	8	1	0	0
0	267	1	10	1	0	0
0	483	1	12	1	0	0
0	1025	1	9	1	0	0

0	350	1	21	1	0	0
0	558	1	5	1	0	0
0	750	0	13	1	0	0
0	900	1	8	1	0	0
0	675	0	10	1	0	0
0	683	0	12	1	0	1
0	833	0	9	1	0	0
0	600	1	7	1	0	0
0	1275	0	5	1	0	0
0	350	1	11	1	1	0
0	717	1	11	1	0	0
0	1025	0	10	1	1	0
0	442	1	8	1	0	0
0	675	1	15	1	0	0
0	992	0	8	1	0	0
0	233	1	9	1	0	0
0	358	0	11	1	0	0
0	642	0	16	1	1	0
0	650	0	9	1	0	0
0	733	0	2	1	0	0
0	975	0	9	1	0	0
0	300	1	10	1	1	0
0	425	0	8	1	0	0
0	642	0	8	1	0	0
0	842	0	8	1	0	0
0	467	1	13	1	0	0
0	900	0	5	1	0	0
0	383	1	5	1	0	0
0	408	1	3	1	0	0
0	800	0	9	1	0	0
0	708	1	16	1	1	0
0	792	0	8	1	0	0
0	983	1	9	1	0	0
0	1033	1	8	1	0	0
0	383	1	9	1	0	0
0	675	0	5	1	0	0
0	1233	1	7	1	0	0
0	258	0	12	1	0	0
0	733	1	6	1	0	0
0	750	1	8	1	0	0
0	100	0	12	1	0	0
0	492	1	10	1	0	0
0	600	1	11	1	0	0
0	517	1	10	1	0	0

0	633	0	8	1	0	0
0	275	1	6	1	0	0
0	483	0	11	1	0	0
0	850	0	5	1	0	0
0	475	0	14	1	0	0
0	508	0	9	1	0	0
0	650	1	10	1	0	0
0	800	0	6	1	0	0
0	858	1	6	1	0	0
0	600	1	7	1	0	0
0	275	1	11	1	0	0
0	633	0	8	1	0	0
0	658	0	7	1	0	0
0	725	0	10	1	0	0
0	775	0	11	1	0	0
0	208	0	9	1	1	0
0	217	0	6	1	0	0
0	667	0	10	1	0	0
0	467	0	9	1	0	0
0	283	0	9	1	0	0
0	692	1	10	1	0	0
0	825	1	10	1	0	0
0	1233	0	8	1	0	1
0	617	0	7	1	0	0
0	1100	0	9	1	1	0
0	200	0	9	1	1	0
0	525	1	16	1	0	0
0	692	0	16	1	0	0
0	642	1	9	1	0	0
0	1117	1	8	1	0	0
0	1050	1	9	1	0	0
0	458	1	13	1	0	0
0	925	0	6	1	0	0
0	217	1	8	1	1	0
0	658	1	8	1	0	0
0	175	0	12	1	0	0
0	692	1	5	1	0	0
0	342	0	10	1	0	0
0	492	1	10	1	0	0
0	142	0	8	1	0	0
0	108	0	10	1	1	0
0	525	0	11	1	0	0
0	733	1	8	1	0	0
0	442	1	14	1	0	0

0	683	0	6	1	0	0
0	892	1	10	1	0	0
0	708	1	6	1	0	0
0	1033	0	6	1	0	0
0	617	0	9	1	0	1
0	850	1	4	1	0	0
0	908	1	8	1	0	0
0	500	1	6	1	0	0
0	842	1	5	1	0	0
0	942	1	7	1	0	0
0	650	0	8	1	0	0
0	775	1	12	1	0	0
0	1108	0	7	1	0	0
0	942	0	4	1	0	1
0	725	1	15	1	0	0
0	667	1	5	1	0	0
0	725	1	12	1	0	0
0	1067	1	7	1	0	0
0	408	1	7	1	0	0
0	692	0	9	1	0	0
0	708	1	8	1	0	0
0	717	0	11	1	0	0
0	333	0	6	1	0	0
0	500	0	6	1	0	0
0	625	1	15	1	0	1
0	658	0	7	1	1	0
0	717	1	3	1	0	0
0	650	0	8	1	0	0
0	625	1	4	1	0	0
0	858	0	7	1	0	0
0	1042	0	10	1	0	0
0	1192	0	9	1	1	0
1	650	0	8	1	0	1
1	483	1	12	1	0	0
1	325	0	10	1	0	0
1	967	1	11	1	0	0
1	558	1	8	1	0	0
1	983	1	11	1	0	0
1	992	0	11	1	0	0
1	533	1	12	1	0	0
1	225	1	7	1	0	0
1	675	1	9	1	0	0
2	633	0	25	1	0	0
2	642	0	15	1	0	0

2	592	1	9	1	1	0
2	600	0	18	1	0	0
2	675	1	7	1	0	0
2	717	1	8	1	0	0
2	475	0	6	1	0	0
2	858	1	18	1	0	0
2	1125	1	14	1	0	1
2	933	0	11	1	0	0
2	983	0	14	1	0	0
2	1250	1	9	1	0	0
2	908	1	16	1	0	0
2	800	1	9	1	0	0
2	725	0	13	1	0	0
2	583	1	9	1	0	0
2	650	1	9	1	0	0
2	617	0	7	1	0	0
2	183	1	11	1	1	0
2	833	1	14	1	0	0
2	333	1	8	1	0	0
2	783	1	10	1	0	0
2	1283	0	5	1	0	0
2	842	0	6	1	0	0
2	1108	0	6	1	0	1
2	617	1	11	1	0	0
2	750	1	9	1	0	0
2	508	1	7	1	0	0
2	1033	1	5	1	0	0
2	675	1	8	1	0	0
2	908	1	5	1	0	0
2	783	0	11	1	0	0
2	600	0	13	1	0	0
2	733	1	8	1	0	1
2	567	0	16	1	0	0
2	058	0	19	1	1	0
2	308	0	6	1	0	0
2	783	1	7	1	1	0
2	692	0	27	1	1	0
2	975	1	5	1	0	0
2	408	0	18	1	0	0
2	642	1	8	1	0	0
2	833	0	7	1	0	0
2	658	1	12	1	0	0
2	825	0	5	1	0	0
2	742	1	13	1	0	0

2	1150	1	7	1	0	0
2	667	0	13	1	0	0
2	817	0	5	1	0	0
2	1008	1	5	1	0	0
2	975	1	10	1	0	0
2	408	1	13	1	0	0
2	1258	0	7	1	0	0
2	792	1	6	1	0	0
2	975	1	10	1	0	0
2	758	1	6	1	0	0
2	733	0	9	1	0	0
2	733	0	7	1	0	0
2	650	1	12	1	0	0
2	292	1	6	1	0	0
2	608	0	7	1	0	0
2	758	0	8	1	0	0
2	825	1	7	1	0	0
2	458	0	14	1	0	0
2	625	1	14	1	0	0
2	683	1	12	1	0	0
2	433	0	14	1	0	0
2	883	0	7	1	0	0
2	625	1	8	1	0	0
2	850	0	11	1	0	0
2	1050	1	5	1	0	0
2	583	1	12	1	0	0
2	242	1	11	1	0	0
2	492	0	14	1	0	1
2	992	1	5	1	0	0
2	392	1	34	1	1	0
2	325	1	7	1	0	0
2	1025	0	7	1	0	0
2	750	0	10	1	1	0
2	617	1	12	1	0	1
2	867	0	8	1	0	0
2	225	0	27	1	1	0
2	692	0	7	1	0	0
2	800	1	10	1	0	0
2	425	1	13	1	0	0
2	975	1	9	1	0	0
2	300	0	8	1	0	0
2	900	1	7	1	0	0
2	750	0	8	1	0	0
2	392	0	10	1	0	0

2	675	0	5	1	0	0
2	1142	1	9	1	0	0
2	550	1	9	1	0	0
2	967	1	8	1	0	0
2	767	1	9	1	0	0
2	775	0	4	1	0	0
2	1142	1	8	1	0	0
2	233	0	8	1	0	0
2	617	0	10	1	0	0
2	833	1	6	1	0	0
2	275	1	7	1	1	1
2	358	0	7	1	1	0
2	775	1	12	1	0	0
2	817	0	11	1	0	0
2	458	0	7	1	0	0
2	875	1	7	1	0	0
2	500	1	10	1	0	0
2	1025	0	8	1	0	0
2	642	0	7	1	0	0
2	068	0	10	1	1	0
2	817	0	8	1	0	0
2	700	1	11	1	0	0
2	508	1	8	1	1	0
2	475	1	10	1	0	0
2	467	0	6	1	0	0
2	317	1	9	1	0	0
2	342	0	9	1	0	0
2	1167	0	9	1	0	0
2	292	0	13	1	0	0
2	450	0	5	1	0	0
2	875	1	7	1	0	0
2	675	0	8	1	0	0
2	550	1	10	1	0	0
2	1033	0	7	1	0	0
2	775	1	7	1	0	0
2	525	0	7	1	1	0
2	658	1	11	1	1	0
2	1033	1	11	1	0	0
2	100	1	6	1	0	0
2	592	1	9	1	0	0
2	358	1	7	1	1	0
2	917	0	14	1	0	0
2	016	0	6	1	0	1
2	1050	0	7	1	0	0

2	908	1	10	1	0	0
2	683	0	6	1	0	0
2	975	0	5	1	0	0
2	567	0	5	1	0	1
2	583	0	7	1	0	0

WBC	HGB	PLT	hs_CRP	ALT	AST	LDH	
46	139	154	64	19	32	269	
146	131	299	04	24	32	386	
164	130	374	16	20	38	311	
61	116	326	205	17	31	322	
66	115	477	04	17	32	0	
69	118	330	183	16	38	523	
62	124	230	104	17	38	342	
54	121	213	88	12	42	384	
58	127	401	892	19	30	303	
65	142	249	53	15	35	366	
61	123	178	246	12	29	241	
119	126	339	04	18	38	291	
156	140	535	04	56	44	278	
93	149	431	04	11	51	523	
135	118	385	132	16	50	448	
5	125	236	249	22	35	298	
58	108	242	58	7	35	332	
6	115	312	101	16	30	346	
48	127	295	12	14	34	401	
51	113	221	134	12	33	279	
62	125	415	45	7	26	323	
125	107	539	04	19	29	391	
53	126	416	13	36	26	220	
117	129	464	214	17	32	0	
105	126	333	61	21	32	302	
301	87	697	176	25	34	337	
87	120	372	43	35	26	304	
64	115	315	206	164	132	583	
65	123	176	186	16	25	190	
75	130	249	18	8	26	297	
123	107	214	174	20	27	255	
69	115	271	117	22	35	431	
81	130	339	14	13	28	307	
66	114	229	77	18	36	290	
55	131	213	19	33	38	473	
95	124	439	04	20	32	393	
61	112	366	6427	22	27	261	
219	120	631	6	20	25	437	
4	108	124	18	212	81	600	
88	130	321	137	21	32	337	
105	110	409	412	35	47	556	
61	136	201	104	17	36	375	
72	121	478	62	14	28	271	

89	119	360	07	6	21	209
66	113	343	171	10	22	299
72	112	250	124	15	31	302
43	112	179	283	64	76	324
44	119	181	27	21	22	222
113	121	449	04	41	36	273
69	114	216	271	24	33	312
102	115	306	236	12	27	243
29	127	224	64	10	30	243
107	133	225	176	25	42	331
9	109	377	49	62	39	509
117	125	547	228	18	24	293
76	123	263	167	24	42	382
79	113	387	389	10	26	329
9	126	305	04	19	23	220
77	123	229	04	17	32	0
55	119	166	121	13	38	383
55	128	257	86	30	42	619
63	126	635	04	17	32	0
103	135	220	31	34	34	306
8	131	233	343	7	22	381
43	113	209	409	22	64	413
75	110	269	252	13	25	271
46	125	356	324	25	31	274
47	124	240	188	13	36	261
82	126	242	175	20	44	341
97	114	271	491	17	24	257
109	135	720	04	11	33	289
78	137	534	04	12	36	343
41	112	231	542	27	47	401
45	114	230	968	10	59	684
89	114	362	282	23	29	284
42	124	202	165	15	34	304
28	117	180	58	7	24	252
91	122	283	49	15	34	346
88	130	581	09	21	26	332
72	129	226	91	16	33	293
11	122	229	442	23	49	365
55	112	583	42	13	24	280
68	111	296	161	16	29	276
122	122	479	15	23	31	382
78	129	244	121	26	39	463
92	120	473	121	11	27	305
171	101	185	1411	18	37	286

62	153	311	04	11	25	297
59	121	537	7	14	25	277
266	117	841	282	120	51	412
7	127	241	278	14	28	337
82	113	375	1389	12	40	603
128	117	359	68	14	30	283
45	121	393	18	17	32	0
74	114	194	65	17	29	287
5	116	286	892	31	41	359
46	112	185	269	16	34	355
6	107	178	996	22	48	401
65	125	210	18	15	33	295
64	126	262	38	10	23	263
136	131	226	39	27	35	357
81	116	221	172	19	36	258
61	136	223	22	32	44	305
89	125	325	96	17	32	415
48	120	147	279	16	30	276
69	116	185	51	22	47	403
61	106	252	19	23	45	447
61	136	171	357	12	25	288
86	133	202	387	20	26	282
58	142	347	218	31	33	388
71	114	260	26	10	25	221
15	134	217	04	126	106	449
129	138	141	23	22	41	333
86	116	208	881	11	29	467
92	121	189	217	17	40	318
118	125	433	229	30	40	420
93	93	421	891	23	22	346
51	123	210	364	23	47	336
58	130	200	155	17	32	0
74	136	259	36	12	30	304
5	113	274	212	6	27	272
57	114	266	09	16	35	284
78	97	202	149	11	55	398
115	132	499	99	17	28	438
52	112	265	11	18	41	513
76	124	228	17	17	32	0
83	130	153	209	28	44	493
103	121	123	04	17	32	0
59	109	220	348	14	29	308
35	131	346	36	12	27	310
43	106	218	198	16	40	384

104	141	473	2	15	35	336
48	112	198	19	17	35	358
88	113	402	682	31	64	621
101	132	420	15	15	20	240
4	103	401	29	28	42	389
54	114	274	81	12	28	246
7	119	208	858	18	29	431
95	123	168	103	16	34	334
122	110	405	169	12	30	322
47	119	214	192	13	30	287
87	120	315	108	17	32	0
56	110	201	496	6	37	345
102	116	284	84	9	21	335
8	125	231	44	46	26	221
124	100	686	131	56	23	391
132	141	526	04	18	23	323
75	121	242	719	17	76	365
47	131	182	199	42	54	434
98	132	341	217	13	37	334
48	138	240	46	13	32	352
88	135	236	264	12	25	284
55	117	373	07	16	18	205
33	113	225	65	13	33	369
65	130	248	802	17	29	397
133	116	395	142	47	81	690
126	128	260	317	19	26	317
81	135	435	7	15	30	255
7	131	303	164	13	27	243
7	120	444	04	37	40	258
63	121	175	173	36	37	258
7	119	295	275	13	30	256
118	139	314	729	15	34	251
68	143	227	4	18	32	319
42	128	204	105	19	47	364
84	135	259	365	19	42	416
41	132	208	49	14	31	273
72	118	457	62	15	34	376
76	137	202	102	37	40	863
62	132	263	286	12	25	273
82	123	259	291	13	28	324
157	119	442	39	23	41	462
72	132	207	40	22	36	272
94	114	247	683	440	467	1251
87	123	328	111	8	35	389

44	117	259	164	15	42	311
58	127	217	746	15	22	321
54	120	209	293	20	31	226
67	133	313	11	12	27	318
122	126	344	239	13	31	441
77	129	195	459	29	43	574
91	123	295	236	31	41	285
67	133	221	146	13	30	328
88	129	220	149	15	20	206
66	113	284	445	17	24	275
43	110	194	637	12	42	327
59	120	314	145	27	35	289
4	106	631	29	81	34	315
57	124	336	101	18	34	399
83	121	443	125	13	27	356
77	120	392	38	15	42	290
65	137	196	132	11	37	323
75	123	253	136	15	40	311
148	119	333	543	13	26	272
78	143	519	54	17	32	0
91	127	306	496	20	25	308
108	134	359	32	28	24	255
73	130	392	13	23	32	226
47	121	243	338	25	38	273
46	130	206	1	15	23	261
47	102	392	47	19	21	252
62	142	333	181	7	22	255
71	128	235	202	19	32	274
925	125	296	606	30	43	411
69	139	397	479	40	46	341
48	128	128	07	48	43	254
117	89	307	25	181	166	541
87	121	264	122	15	39	304
106	101	497	31	34	31	316
293	126	430	47	34	46	318
66	120	281	186	17	32	0
06	67	173	972	18	15	147
75	112	202	225	18	22	312
89	128	204	289	21	40	312
65	128	394	99	20	32	238
625	126	335	212	21	26	244
132	128	471	371	26	26	410
102	124	644	04	17	30	255
8	100	520	18	25	36	259

73	121	280	392	14	20	205
75	117	258	1018	24	57	836
86	120	322	57	13	20	196
49	119	238	04	28	34	327
123	127	392	363	35	50	372
10	137	254	115	20	30	377
34	132	202	293	11	16	237
79	131	147	215	25	32	315
56	103	353	68	30	39	466
11	124	306	21	13	30	401
99	112	192	04	23	28	258
61	120	249	9	22	37	284
71	112	215	438	44	40	343
5	122	109	439	15	36	305
67	138	290	04	17	32	0
57	117	217	297	24	40	289
43	108	447	133	11	28	343
98	108	153	88	12	38	335
73	113	214	1117	30	47	318
74	127	282	147	15	27	300
76	121	407	04	19	25	247
85	115	183	86	24	37	296
68	135	322	22	14	40	379
66	128	191	414	17	47	759
74	131	251	709	22	27	264
64	116	421	09	27	30	263
93	89	221	102	14	42	470
52	130	258	287	12	24	216
109	140	364	04	17	29	381
143	96	225	66	13	15	225
57	121	261	45	11	45	346
74	129	425	129	22	29	301
7	126	241	76	22	30	244
58	126	248	12	13	27	324
63	134	231	31	29	42	319
96	99	232	116	15	62	659
85	124	353	233	13	33	399
69	116	341	581	9	30	368
224	102	471	989	18	38	440
96	125	388	07	14	23	252
111	130	359	412	34	66	656
48	131	275	08	9	23	300
58	129	338	145	86	80	427
31	108	326	193	11	24	222

68	118	208	373	13	30	301
58	118	291	229	10	18	230
51	118	541	04	13	27	304
136	136	441	04	34	23	294
108	102	545	297	6	12	223
571	137	225	51	20	35	306
55	118	247	529	14	21	310
112	133	221	71	13	50	406
49	131	190	225	15	31	299
56	123	187	41	11	19	225
83	121	199	416	13	18	203
83	117	334	151	43	55	437
83	125	213	128	17	42	387
64	126	251	83	21	31	282
115	127	311	339	12	27	278
61	104	210	184	10	25	233
68	105	328	07	13	27	328
692	114	315	403	26	19	230
59	121	274	59	24	45	381
61	109	487	14	38	47	241
134	129	287	24	29	28	237
64	123	208	106	13	32	286
85	135	277	134	13	28	383
114	67	254	22	12	39	582
71	119	161	82	25	45	488
55	110	415	42	16	36	297
8	127	298	09	17	32	0
6	149	142	164	11	20	224
74	103	387	3	21	33	254
722	123	275	12	11	26	385
47	113	320	125	23	27	269
83	114	308	73	12	22	288
47	120	172	82	35	65	649
52	128	403	18	14	19	359
53	131	357	69	12	29	292
6	110	211	41	24	52	399
68	131	254	144	21	42	394
56	115	151	1473	15	34	339
79	117	265	34	23	46	344
71	132	264	468	20	35	325
77	117	257	629	17	32	0
14	111	446	574	164	27	343
85	105	358	92	26	41	363
74	138	319	135	16	26	273

75	129	273	18	29	40	451
108	118	356	97	6	27	273
89	138	303	04	22	35	450
74	126	219	28	19	37	346
10	124	432	19	20	41	295

Consolidatior	Lung_exudati	Pleural_thick	Pleural_effus	lung_patch	mosaic_signs	bronchial_oc
1	0	0	0	0	0	0
1	0	0	0	1	0	0
0	0	1	0	1	0	0
1	0	0	0	1	0	0
1	0	0	0	1	0	0
1	0	0	1	1	0	0
1	0	0	0	1	0	0
1	0	1	0	1	0	0
1	0	0	0	1	0	0
0	0	0	0	1	0	0
1	0	0	1	0	0	0
1	0	0	0	1	0	0
0	0	1	0	1	0	0
0	0	0	0	1	0	0
0	0	1	0	1	0	0
1	0	0	0	0	0	0
1	0	0	0	0	0	0
1	0	0	0	1	0	0
1	0	0	0	1	0	0
1	0	0	0	1	0	0
0	0	1	0	1	0	0
1	0	0	0	0	0	0
0	0	1	1	1	0	0
0	0	0	0	1	0	0
0	0	0	0	1	0	0
1	0	1	1	0	0	0
1	0	0	1	0	0	0
1	0	1	1	1	0	0
1	0	0	0	1	0	0
1	0	1	0	1	0	0
0	0	0	0	1	1	0
0	0	0	0	1	0	0
1	0	0	0	1	0	0
1	0	0	0	1	0	0
1	0	0	0	0	0	0
0	0	0	0	1	0	0
1	0	0	0	0	0	0
1	0	0	1	1	0	0
1	0	1	1	1	0	0
0	0	1	0	1	0	0
1	0	0	0	0	0	0
1	0	0	0	1	0	0
0	0	0	0	1	0	0

1	0	0	0	1	0	0
1	0	0	0	1	0	0
1	0	0	0	1	0	0
1	0	0	0	1	0	0
1	0	0	0	1	0	0
1	0	0	1	1	0	0
1	0	1	0	0	0	0
1	0	0	0	1	0	0
1	0	0	0	0	0	0
0	0	1	1	1	0	0
1	0	0	1	1	0	0
1	0	0	1	0	0	1
0	0	0	0	1	0	0
1	0	1	1	0	0	0
1	0	0	0	1	0	1
1	0	0	1	0	0	0
1	0	1	1	0	0	0
1	0	0	0	1	0	0
0	0	0	0	1	0	0
1	1	0	0	0	0	0
1	0	0	1	1	0	0
0	0	0	1	1	0	0
1	0	0	0	1	0	0
1	0	1	0	1	0	0
0	0	0	0	1	0	0
1	0	0	0	1	0	0
1	0	0	0	0	0	0
1	0	0	0	0	0	0
1	0	0	0	0	0	0
1	0	0	0	1	0	0
1	0	0	1	1	0	0
1	0	0	1	1	0	0
0	0	0	0	1	0	0
1	0	1	0	1	0	1
0	0	0	0	1	0	0
0	0	0	0	1	0	0
1	0	0	0	1	0	0
1	0	0	0	1	0	0
1	0	1	0	0	0	0
1	0	1	0	1	0	0
0	0	1	0	1	0	0
0	0	0	0	1	0	0
1	0	1	1	1	0	0
1	0	0	0	1	0	0

1	0	0	0	1	0	0
1	0	0	0	0	0	0
1	0	0	1	1	0	0
1	0	1	0	1	0	0
1	0	0	1	0	0	0
1	0	1	0	1	0	0
1	0	0	0	0	0	0
0	0	0	0	1	0	0
0	0	0	1	1	0	0
1	0	0	0	0	0	0
1	0	1	0	0	0	0
0	0	0	0	1	0	0
1	0	0	0	1	0	0
0	0	0	0	1	0	0
1	0	0	0	1	0	1
0	0	0	0	1	0	0
1	0	1	0	1	0	0
1	0	0	1	0	0	0
0	0	0	0	1	0	0
1	0	0	0	1	0	0
0	0	0	0	1	0	0
1	0	0	0	1	0	0
0	1	0	0	1	0	0
0	0	1	0	1	0	0
0	0	0	0	1	1	0
0	0	0	0	1	1	0
1	0	0	1	1	0	0
1	0	0	1	1	0	0
1	0	1	0	1	0	0
0	0	0	0	1	0	0
1	0	0	0	1	0	0
1	0	0	0	1	0	0
1	0	0	0	1	0	0
0	0	0	0	1	0	0
0	0	0	0	1	0	0
1	0	0	0	1	0	0
1	0	1	0	0	0	0
1	0	0	1	0	0	0
1	0	1	0	1	0	0
1	0	0	0	1	0	0
0	0	0	0	1	0	0
1	0	0	0	1	0	0
0	0	1	0	1	0	0
1	0	0	1	0	0	0

1	0	0	1	0	0	0
1	0	0	1	1	0	0
1	0	0	0	0	0	0
1	0	0	0	0	0	0
0	0	0	0	1	0	0
0	0	0	0	1	0	0
1	0	1	0	1	0	0
0	0	0	0	1	0	0
0	0	0	1	0	0	0
1	0	0	0	1	0	0
0	0	0	1	0	0	0
1	0	0	0	1	0	0
0	0	0	0	1	0	0
0	0	0	0	1	0	0
1	0	1	1	0	0	0
0	0	0	0	1	0	0
1	0	0	0	1	0	0
1	0	1	0	1	0	0
1	0	0	0	0	0	0
1	0	0	0	0	0	0
1	0	1	0	0	0	0
1	0	0	0	0	0	0
1	0	0	0	0	0	1
1	0	0	0	0	0	0
1	0	0	0	1	0	1
1	0	0	0	1	0	0
1	0	0	0	0	0	0
1	0	0	0	1	0	0
1	0	0	0	0	0	0
0	0	0	0	1	0	0
0	0	0	0	1	0	0
0	0	0	0	1	1	0
0	0	0	0	1	0	0
1	0	1	0	0	0	0
1	0	1	0	0	0	0
1	0	1	0	0	0	0
1	0	0	0	1	0	0
1	0	1	0	0	0	0
0	0	0	0	1	0	0
1	0	0	0	1	0	0
1	0	0	0	1	0	0
1	0	1	1	0	0	0
1	0	1	1	1	0	0
1	0	0	0	0	0	0

1	0	0	0	1	0	0
0	0	0	0	1	1	0
1	0	0	0	1	0	0
1	0	0	0	1	0	0
1	0	0	0	1	0	0
1	0	1	0	0	0	0
1	0	1	0	1	0	0
1	0	0	0	0	0	0
0	0	0	0	1	0	0
1	0	0	1	0	0	0
1	0	0	1	0	0	0
1	0	0	0	0	0	0
1	0	0	1	0	0	0
1	0	1	0	0	0	0
0	0	0	0	1	0	0
0	0	0	0	1	0	0
0	0	0	0	1	1	0
1	0	1	0	0	0	0
0	0	0	0	1	0	0
0	0	0	1	1	0	0
0	0	0	1	1	0	0
1	0	0	0	0	0	1
0	0	1	1	1	0	0
1	0	1	0	0	0	0
0	0	0	0	1	0	0
1	0	0	0	1	0	0
1	0	0	0	1	0	0
0	0	0	1	1	0	0
1	0	0	0	1	0	0
1	0	0	0	1	0	0
0	0	0	0	1	1	0
0	0	0	0	1	0	0
1	0	1	0	1	0	0
1	0	1	0	0	0	0
1	0	0	0	0	0	0
1	0	0	1	0	0	0
1	0	0	1	0	0	0
1	0	0	0	0	0	0
1	0	0	0	0	0	0
1	0	0	1	1	0	0
1	0	0	0	1	0	0
0	0	0	1	1	0	0
0	0	1	1	0	0	0
0	0	0	0	1	0	0

0	0	0	0	1	0	0
1	0	1	0	0	0	0
0	0	0	0	1	0	0
0	0	1	0	1	0	0
1	0	0	0	0	0	0
1	0	1	0	1	0	0
1	1	1	0	0	0	0
1	0	0	0	0	0	0
1	0	1	1	1	0	0
0	0	1	0	1	0	0
1	0	0	0	0	0	0
1	0	0	1	0	0	0
1	0	0	0	1	0	0
1	0	0	0	1	0	0
1	0	0	0	0	0	0
1	0	0	1	0	0	0
1	0	0	1	0	0	0
1	0	1	1	1	0	0
0	0	0	0	1	0	0
1	0	1	0	0	0	0
1	0	0	0	1	0	0
1	0	0	0	1	0	0
1	0	0	0	1	0	0
1	0	1	0	0	0	0
1	0	0	0	1	0	0
1	0	0	1	1	0	0
1	0	1	0	1	0	0
0	0	1	0	1	0	0
1	0	1	1	0	0	0
0	0	1	0	1	0	0
1	0	1	0	1	0	1
0	0	0	0	1	0	0
0	0	0	1	1	0	0
1	0	0	0	0	0	0
1	0	0	0	1	0	0
1	0	1	1	0	0	0
1	0	0	0	1	0	0
1	0	0	0	1	0	0
1	0	0	1	1	0	0
0	0	0	0	1	0	0
1	0	0	0	1	0	0
1	0	0	1	0	0	0
1	0	0	0	1	0	0
1	0	1	0	1	0	0

1	0	0	0	1	0	0
1	0	0	0	1	0	0
0	0	0	0	1	0	0
0	0	1	1	1	0	0
1	0	0	1	1	0	0
0	0	0	0	1	0	0
1	0	1	1	0	0	0
1	0	0	0	1	0	0
1	0	0	0	1	0	0
1	0	0	0	1	0	0
0	0	0	0	1	1	0
1	0	0	0	1	0	0
1	0	1	0	1	0	0
1	0	0	0	0	0	0
1	0	1	0	0	0	0
1	0	0	0	0	0	0
1	0	1	0	1	0	0
1	0	0	1	1	0	0
1	0	1	0	0	0	0
0	0	1	0	1	0	0
1	0	0	0	0	0	0
1	0	0	0	1	0	0
1	0	0	0	1	0	0
1	0	0	0	0	0	0
0	0	0	0	1	0	0
1	0	0	0	1	0	0
1	0	0	0	0	0	0
1	0	1	0	1	0	0
1	1	0	0	0	0	0
1	0	0	0	1	0	0
1	0	0	0	1	0	0
1	0	0	0	0	0	0
1	0	0	1	0	0	0
1	0	0	0	0	0	0
0	0	0	0	1	0	0
0	0	0	0	1	0	0
0	0	0	0	1	1	0
1	0	0	1	1	0	0
1	0	0	0	1	1	0
1	0	0	0	1	0	1
1	0	0	0	1	0	0
1	0	0	1	0	0	0
0	0	0	0	1	0	0
1	0	0	1	0	0	0

0	0	0	1	1	0	0
1	0	0	0	1	0	0
1	0	0	1	0	0	0
0	0	0	0	1	0	0
1	0	0	0	1	0	0

fever	Duration_of_	Duration_of_	Aci_onset_tir	Aci_usage_tir	Dox_onset_ti	Dox_usage_ti
1	1	11	1	17	0	0
1	1	10	1	13	0	0
1	1	6	1	6	0	0
1	1	4	1	6	0	0
1	1	4	1	11	0	0
1	2	6	2	8	0	0
1	2	6	2	11	0	0
1	2	5	2	7	0	0
1	2	5	2	4	0	0
1	2	4	2	12	0	0
1	2	4	2	9	0	0
1	2	2	2	10	0	0
0	0	0	2	8	0	0
1	3	6	3	6	0	0
1	3	6	3	12	0	0
1	3	6	3	10	0	0
1	3	4	3	9	0	0
1	3	4	3	6	0	0
1	3	4	3	11	0	0
1	3	4	3	8	0	0
1	3	0	3	6	0	0
1	3	0	3	5	0	0
1	3	0	3	6	0	0
1	4	9	4	16	0	0
1	4	6	4	6	0	0
1	4	6	4	10	0	0
1	4	6	4	8	0	0
1	4	6	4	12	0	0
1	4	5	4	9	0	0
1	4	4	4	7	0	0
1	4	3	4	5	0	0
1	4	3	4	3	0	0
1	4	1	4	10	0	0
1	4	1	4	11	0	0
1	4	1	4	3	0	0
1	5	6	5	9	0	0
1	5	6	5	12	0	0
1	5	5	5	9	0	0
1	5	5	5	8	0	0
1	5	5	5	8	0	0
1	5	4	5	11	0	0
1	5	4	5	9	0	0
1	5	4	5	11	0	0

1	5	3	5	10	0	0
1	5	3	5	15	0	0
1	5	3	5	13	0	0
1	5	3	5	6	0	0
1	5	2	5	8	0	0
1	5	2	5	14	0	0
1	5	2	5	8	0	0
1	5	1	5	8	0	0
1	5	1	5	3	0	0
1	5	0	5	8	0	0
1	5	0	5	9	0	0
0	0	0	5	5	0	0
1	6	4	6	9	0	0
1	6	4	6	8	0	0
1	6	3	6	15	0	0
1	6	2	6	10	0	0
1	6	2	6	3	0	0
1	6	2	6	3	0	0
1	6	2	6	10	0	0
1	6	2	6	5	0	0
1	6	2	6	6	0	0
1	6	1	6	4	0	0
1	6	0	6	11	0	0
1	6	0	6	6	0	0
1	6	0	6	6	0	0
1	5	0	6	7	0	0
1	4	0	6	5	0	0
1	7	3	7	10	0	0
1	7	3	7	5	0	0
1	7	3	7	8	0	0
1	7	2	7	5	0	0
1	7	2	7	5	0	0
1	7	2	7	7	0	0
1	7	2	7	11	0	0
1	7	1	7	8	0	0
1	7	1	7	6	0	0
1	7	1	7	5	0	0
1	7	0	7	9	0	0
1	7	0	7	9	0	0
1	7	0	7	6	0	0
1	6	0	7	16	0	0
1	6	0	7	10	0	0
1	5	0	7	6	0	0
1	2	0	7	10	0	0

1	1	0	7	11	0	0
1	8	5	8	10	0	0
1	8	4	8	5	0	0
1	8	4	8	6	0	0
1	8	3	8	6	0	0
1	8	3	8	5	0	0
1	8	3	8	8	0	0
1	8	2	8	5	0	0
1	8	2	8	6	0	0
1	8	0	8	16	0	0
1	7	0	8	8	0	0
1	7	0	8	10	0	0
1	7	0	8	8	0	0
1	7	0	8	5	0	0
1	6	0	8	9	0	0
1	5	0	8	6	0	0
1	5	0	8	8	0	0
1	4	0	8	6	0	0
1	9	3	9	6	0	0
1	9	0	9	10	0	0
1	9	0	9	10	0	0
1	9	0	9	7	0	0
1	9	0	9	7	0	0
1	6	0	9	9	0	0
1	3	0	9	6	0	0
0	0	0	9	9	0	0
1	10	1	10	13	0	0
1	10	1	10	10	0	0
1	10	0	10	11	0	0
1	9	0	10	5	0	0
1	8	0	10	3	0	0
1	7	0	10	10	0	0
1	7	0	10	3	0	0
1	6	0	10	9	0	0
1	6	0	10	6	0	0
1	3	0	10	8	0	0
1	11	2	11	5	0	0
1	9	0	11	6	0	0
1	9	0	11	6	0	0
1	11	0	12	10	0	0
1	10	0	12	15	0	0
1	9	0	12	11	0	0
1	9	0	12	8	0	0
1	7	0	12	3	0	0

1	7	0	12	8	0	0
1	7	0	12	6	0	0
1	13	1	13	5	0	0
1	12	0	13	6	0	0
1	10	0	13	8	0	0
1	10	0	13	11	0	0
1	10	0	13	8	0	0
1	9	0	13	6	0	0
1	9	0	13	5	0	0
1	9	0	13	3	0	0
1	8	0	13	6	0	0
1	8	0	13	17	0	0
1	7	0	13	13	0	0
1	12	0	14	8	0	0
1	11	0	14	8	0	0
1	10	0	14	6	0	0
1	7	0	14	8	0	0
1	7	0	14	3	0	0
1	11	0	15	5	0	0
1	8	0	15	10	0	0
1	4	0	15	3	0	0
1	16	1	16	5	0	0
1	7	0	16	10	0	0
1	7	0	16	3	0	0
1	11	0	17	6	0	0
1	3	0	17	9	0	0
1	9	0	19	10	0	0
1	5	0	19	11	0	0
1	11	0	21	6	0	0
1	8	0	21	6	0	0
1	8	0	24	6	0	0
1	3	0	26	9	0	0
1	3	5	0	0	3	10
1	4	1	0	0	4	10
1	7	3	0	0	7	10
1	8	2	0	0	8	10
1	9	1	0	0	9	10
1	5	0	0	0	9	6
1	5	0	0	0	11	10
1	8	0	0	0	13	10
1	14	1	0	0	14	10
1	6	0	0	0	18	10
1	1	14	1	3	5	10
1	4	3	4	1	5	10

1	1	5	1	3	6	10
1	2	5	2	2	6	10
1	2	5	2	3	6	14
1	3	4	3	3	6	10
1	4	8	4	3	6	10
1	5	1	5	2	6	10
1	5	0	5	3	6	7
1	1	8	1	5	7	10
1	1	4	1	5	7	14
1	3	5	3	3	7	0
1	4	0	4	3	7	10
1	5	5	5	2	7	10
1	5	0	5	3	7	10
1	7	0	8	1	7	10
1	2	5	2	6	8	14
1	4	12	4	4	8	10
1	4	3	4	3	8	14
1	4	2	4	5	8	10
1	5	5	5	3	8	10
1	5	2	5	3	8	12
1	5	2	5	2	8	10
1	6	8	6	3	8	10
1	6	3	6	1	8	10
1	6	2	6	3	8	10
1	6	2	6	3	8	10
1	6	1	6	2	8	10
1	6	0	6	3	8	10
1	2	7	2	3	9	10
1	2	6	2	3	9	10
1	2	5	2	5	9	7
0	0	0	3	5	9	10
1	4	12	4	5	9	10
1	4	3	4	5	9	10
1	4	0	4	11	9	2
1	5	9	5	5	9	12
1	5	3	5	3	9	10
1	6	15	6	2	9	10
1	6	2	6	3	9	7
1	6	0	6	3	9	10
1	2	0	6	3	9	10
1	7	10	7	2	9	10
1	7	7	7	3	9	10
1	5	0	7	2	9	10
1	8	5	8	2	9	10

1	8	0	8	2	9	10
1	6	0	8	2	9	10
1	2	6	2	5	10	10
1	4	8	4	3	10	10
1	4	7	4	5	10	10
1	4	4	4	3	10	10
1	4	4	4	3	10	10
1	4	2	4	5	10	10
1	5	5	5	6	10	10
1	5	4	5	4	10	10
1	5	0	5	3	10	10
1	6	2	6	3	10	7
1	6	1	6	3	10	10
1	6	0	6	3	10	10
1	7	13	7	1	10	10
1	7	2	7	3	10	12
1	7	1	7	3	10	10
1	8	3	8	2	10	12
1	8	2	8	3	10	10
1	6	0	8	2	10	12
1	5	0	8	2	10	10
1	5	0	8	2	10	10
1	9	0	9	1	10	10
1	8	0	9	2	10	10
1	10	0	14	3	10	10
1	3	0	3	6	11	9
1	5	5	5	5	11	10
1	5	3	5	5	11	10
1	6	7	6	5	11	10
1	7	12	7	3	11	10
1	7	2	7	5	11	10
1	8	1	8	3	11	10
1	7	0	8	3	11	10
1	4	0	8	3	11	10
0	0	0	8	3	11	10
1	9	10	9	1	11	10
1	9	1	9	3	11	10
1	9	1	9	3	11	10
1	9	0	9	2	11	10
1	10	1	10	5	11	10
1	3	5	3	3	12	10
1	3	4	3	3	12	10
1	4	3	4	12	12	7
1	5	3	5	4	12	7

1	7	3	7	5	12	10
1	7	3	7	6	12	10
1	7	1	7	5	12	7
1	8	3	8	4	12	10
1	9	7	9	3	12	10
1	9	1	9	3	12	10
1	3	5	3	3	14	6
1	4	2	4	8	14	10
1	5	1	5	5	14	10
1	6	3	6	3	14	10
1	5	0	6	5	14	10
1	5	0	6	5	14	10
1	7	3	7	3	14	10
1	8	0	8	6	14	10
1	6	0	9	5	14	10
1	10	0	10	5	14	14
1	3	2	3	6	15	10
0	0	0	7	4	15	10
1	8	5	8	6	15	10
0	0	0	10	5	15	10
1	8	0	11	3	15	10
1	7	0	12	3	15	10
1	6	0	14	2	15	10
1	3	5	3	6	16	14
1	8	0	8	5	16	10
1	7	0	12	5	16	10
1	5	0	9	3	17	10
1	5	0	9	6	17	10
1	3	0	10	6	17	10
1	5	11	5	7	18	10
1	5	2	5	7	18	10
1	3	0	6	3	18	10
1	10	2	10	3	18	10
1	13	0	14	4	18	10
1	2	3	2	8	19	10
1	9	0	9	8	19	10
1	8	0	9	9	19	10
1	6	0	10	5	19	7
1	4	0	17	3	19	10
1	4	2	4	11	20	7
1	5	0	10	5	20	10
1	12	0	17	3	20	10
1	2	7	2	5	22	14
1	8	0	12	6	23	10

1	13	0	19	3	23	10
1	7	0	24	3	26	10
1	8	0	9	8	31	7
1	6	0	8	10	33	10
1	6	3	6	12	35	10

hor_onset_ti	hormone	oxygen_thera	electronic_br	gg
0	0	1	0	0
10	1	1	1	1
0	0	1	1	0
0	0	1	1	0
0	0	1	1	0
1	1	0	0	0
0	0	0	0	0
0	0	1	1	0
0	0	1	1	0
0	0	1	1	0
0	0	1	1	0
0	0	1	1	0
0	0	1	1	0
0	0	1	1	0
0	0	1	1	0
0	0	0	0	0
0	0	1	1	0
0	0	1	1	0
1	1	1	1	0
7	1	1	1	0
0	0	0	1	0
0	0	0	0	0
0	0	0	1	0
0	0	1	1	1
4	1	1	1	0
0	0	1	1	0
0	0	1	1	0
0	0	1	1	0
7	1	0	0	0
0	0	0	1	0
8	1	1	1	0
0	0	2	0	1
0	0	1	1	0
0	0	1	1	0
0	0	0	0	0
13	1	1	0	0
0	0	1	1	0
0	0	1	1	0
0	0	1	1	0
0	0	1	1	0
0	0	2	1	0
0	0	1	1	0
0	0	1	1	0

0	0	1	1	0
0	0	1	1	0
0	0	1	1	0
0	0	1	1	0
0	0	1	1	0
0	0	0	0	0
0	0	1	1	0
6	1	2	1	0
0	0	1	1	0
0	0	1	1	0
0	0	0	0	0
0	0	1	1	0
1	1	1	0	0
0	0	0	0	0
0	0	1	1	0
0	0	1	1	0
10	1	2	1	0
0	0	0	0	0
0	0	1	1	1
7	1	0	0	0
0	0	1	1	0
0	0	1	1	0
0	0	1	1	0
0	0	0	0	0
6	1	1	1	0
7	1	0	1	0
0	0	1	1	0
0	0	1	1	0
0	0	0	1	0
0	0	0	0	0
0	0	2	1	0
0	0	0	0	0
5	1	2	0	1
0	0	1	1	0
0	0	1	1	0
0	0	0	0	0
7	1	1	0	0
0	0	1	1	0
0	0	1	1	0
0	0	1	1	0
0	0	1	1	0
16	1	0	0	1
0	0	1	1	0
0	0	1	1	0

0	0	1	1	0
8	1	1	1	0
0	0	1	1	0
0	0	1	1	0
0	0	1	1	0
0	0	1	1	0
0	0	1	1	0
0	0	0	0	0
0	0	1	1	0
0	0	1	1	0
0	0	1	1	0
0	0	1	1	0
0	0	1	1	0
0	0	1	1	0
0	0	1	1	0
0	0	1	1	0
0	0	1	1	0
0	0	1	1	0
1	1	0	0	0
0	0	1	1	0
0	0	0	0	0
1	1	0	0	0
10	1	1	0	0
0	0	1	0	0
0	0	1	1	0
0	0	1	1	0
0	0	1	1	0
0	0	0	0	0
0	0	0	0	0
0	0	1	1	0
0	0	0	0	0
0	0	0	0	0
0	0	0	0	0
0	0	1	1	0
0	0	1	1	0
10	1	1	1	0
0	0	1	1	0
0	0	1	1	0
0	0	1	0	0
0	0	1	1	0
0	0	1	1	0
0	0	0	0	0
0	0	1	1	0
0	0	0	1	0

0	0	1	1	0
7	1	0	0	1
0	0	1	1	0
0	0	1	1	0
0	0	1	1	0
0	0	1	1	0
0	0	1	1	0
0	0	1	1	0
0	0	1	1	0
0	0	1	1	0
0	0	1	1	0
0	0	1	1	0
0	0	1	1	0
1	1	1	0	1
0	0	2	1	0
6	1	1	1	0
0	0	0	0	0
0	0	1	1	0
0	0	0	1	0
0	0	0	0	0
0	0	0	0	0
0	0	1	1	0
0	0	1	1	0
1	1	0	0	0
0	0	1	1	0
0	0	1	1	0
5	1	2	1	1
29	1	1	1	0
0	0	1	1	0
0	0	1	1	0
36	1	1	1	0
0	0	1	1	0
0	0	0	0	0
1	1	2	0	0
0	0	2	1	0
0	0	0	0	0
0	0	1	1	0
0	0	0	0	0
0	0	0	0	0
0	0	1	1	0
0	0	1	1	0
18	1	0	0	0
10	1	1	1	0
1	1	2	1	0

6	1	0	0	0
0	0	1	1	0
1	1	1	1	0
1	1	1	1	0
0	0	1	1	0
6	1	1	0	0
0	0	0	0	0
0	0	1	0	0
1	1	1	1	0
0	0	0	0	0
0	0	0	0	0
0	0	0	0	0
0	0	1	1	1
6	1	1	1	0
10	1	2	0	0
7	1	2	1	0
0	0	1	1	0
7	1	2	0	0
0	0	1	1	0
6	1	1	1	0
0	0	1	1	0
6	1	1	1	0
0	0	2	0	0
0	0	0	0	0
7	1	0	0	0
9	1	0	1	0
8	1	0	0	0
0	0	1	1	0
0	0	0	0	0
0	0	1	1	0
0	0	1	1	0
0	0	1	1	0
1	1	2	0	0
9	1	1	1	0
12	1	1	1	0
10	1	1	1	0
10	1	1	1	0
0	0	0	0	0
0	0	0	1	0
0	0	0	0	0
0	0	0	0	0
0	0	0	0	0
12	1	1	1	1
0	0	0	0	0

8	1	1	1	0
0	0	0	0	0
0	0	0	0	0
0	0	0	0	0
0	0	0	0	0
0	0	0	1	0
0	0	0	0	0
10	1	1	1	0
0	0	1	1	0
0	0	1	1	0
14	1	0	0	0
1	1	0	0	0
10	1	0	0	0
0	0	1	1	0
9	1	1	1	0
12	1	1	1	1
0	0	0	0	0
0	0	0	1	0
0	0	0	0	0
0	0	0	1	0
0	0	1	1	0
0	0	0	0	0
10	1	2	1	0
0	0	1	0	0
12	1	2	0	1
0	0	0	0	0
0	0	1	1	0
1	1	1	0	0
0	0	0	0	0
0	0	1	1	0
0	0	1	1	0
0	0	0	1	0
0	0	1	1	0
7	1	0	0	0
0	0	1	1	0
11	1	1	1	0
0	0	0	0	0
0	0	0	0	0
5	1	0	1	0
14	1	2	1	1
0	0	1	1	0
0	0	0	0	0
0	0	2	1	0
0	0	1	1	0

9	1	2	1	0
0	0	0	0	0
0	0	1	1	0
0	0	0	0	0
0	0	1	1	0
0	0	0	0	0
0	0	0	0	0
1	1	1	1	0
0	0	1	0	0
0	0	1	0	0
0	0	1	1	0
0	0	1	1	0
0	0	0	0	0
0	0	1	1	0
1	1	1	0	0
0	0	1	1	0
0	0	1	1	0
7	1	0	0	0
16	1	1	1	0
0	0	1	1	0
0	0	0	0	0
15	1	1	1	1
0	0	0	0	0
3	1	0	1	0
0	0	1	1	0
16	1	1	1	0
0	0	1	1	0
12	1	0	0	0
0	0	1	1	0
0	0	1	1	0
15	1	1	1	0
12	1	1	1	0
0	0	1	1	0
0	0	1	1	0
0	0	1	1	0
16	1	0	0	0
0	0	1	1	0
11	1	1	1	0
0	0	1	1	0
0	0	1	1	0
20	1	1	1	0
17	1	1	1	0
17	1	2	1	1
0	0	1	1	0

23	1	2	1	0
0	0	0	0	0
9	1	1	1	0
0	0	0	0	0
0	0	1	1	0
